# Preserving the Mucosa to the Maximum Possible Extent for Endoscopic Submucosal Dissection of Subcircumferential Superficial Esophageal Carcinoma

**DOI:** 10.1155/2018/3540204

**Published:** 2018-04-23

**Authors:** Masaya Uesato, Kentarou Murakami, Yoshihiro Nabeya, Kazunori Fugo, Hisahiro Matsubara

**Affiliations:** ^1^Department of Frontier Surgery, Chiba University Graduate School of Medicine, Chiba-shi, Chiba 260-8670, Japan; ^2^Division of Gastroenterological Surgery, Chiba Cancer Center, Chiba 260-8717, Japan; ^3^Department of Molecular Pathology, Chiba University Graduate School of Medicine, Chiba-shi, Chiba 260-8670, Japan

## Abstract

**Aim:**

To show our unique strategy of endoscopic submucosal dissection (ESD) for esophageal squamous cell carcinoma larger than the subcircumference.

**Methods:**

From April 2011, we used a mucosal preservation method called the log bridge (LB) method for the lesion larger than the subcircumference. The patients in whom the circumference of the mucosal defect was 5/6 to <1 were classified into the LB group; those who underwent whole circumferential ESD were classified into the non-LB group. The data were collected retrospectively and were compared between the two groups.

**Results:**

Eighteen patients into the LB group and 7 into the non-LB group were classified. The median number of endoscopic balloon dilation sessions after ESD in the LB group tended to be lower than that in the non-LB group. The mean period until complete epithelialization after ESD was significantly shorter in the LB group. The rates of curative resection were 100% (7/7) in the non-LB group and 61.1% (11/18) in the LB group. However, there was no local recurrence in either group for approximately two years.

**Conclusion:**

In cases involving subcircumferential esophageal lesions, the LB method is useful for achieving rapid healing and might be related to a reduced degree of esophageal stricture.

## 1. Introduction

Endoscopic submucosal dissection (ESD) is a useful and minimally invasive procedure that is used in the management of early esophageal cancer. It also enables the en bloc resection of large lesions [1, 2]. Moreover, ESD facilitates the accurate histopathological assessment of specimens resected en bloc with tumor-free horizontal/vertical margins, thereby resulting in the prevention of residual disease and local recurrence [3, 4]. However, extensive resection results in esophageal stricture or slow healing. The frequency of stricture after ESD for esophageal cancer in high-risk patients (a mucosal defect of >3/4 of the circumference) is approximately 70–90% [5–7]. However, the intraregional injection of steroids or the oral administration of prednisolone may be able to prevent stricture after ESD [8, 9]. Our institution administers multiple intraregional steroid injections (mISI) in the first session (just after ESD) and every two weeks to effectively suppress inflammation. Among the patients who underwent ESD without ISI at our institution from December 2002 to March 2011, the rate of stricture after ESD in patients with a wound of <1/2 the circumference was 0% (0/98 patients), while the rate in patients with a lesion covering < 1/2 the circumference was 7.62% (8/105 patients) (data not shown). Thus, the method of preserving the mucosa, the so-called log bridge (LB) method, which systematically preserves the mucosa to the maximum possible extent, is performed for subcircumferential superficial esophageal squamous cell carcinoma. The aim of this retrospective study is to prove the usefulness of the LB method in promoting rapid healing and preventing stricture after ESD.

## 2. Materials and Methods

### 2.1. Patients

A total of 104 patients were treated with ESD plus ISI for esophageal carcinoma at the Chiba University Graduate School of Medicine from April 2011 to February 2018. The LB method was attempted for lesions larger than the subcircumference. As a result, the patients in whom the mucosa was preserved and the circumference of the mucosal defect was 5/6 to <1 were classified into the LB group; those who underwent whole circumferential ESD were classified into the non-LB group. Patients in whom the esophageal carcinoma was located in the cervical or abdominal esophagus, where stenosis is likely to occur, were excluded. Data from these patients were collected retrospectively. The management of wound healing and the pathological evaluation of the resected specimen were examined in the two groups.

### 2.2. ESD with the Log Bridge (LB) Method

**Figure 1:**
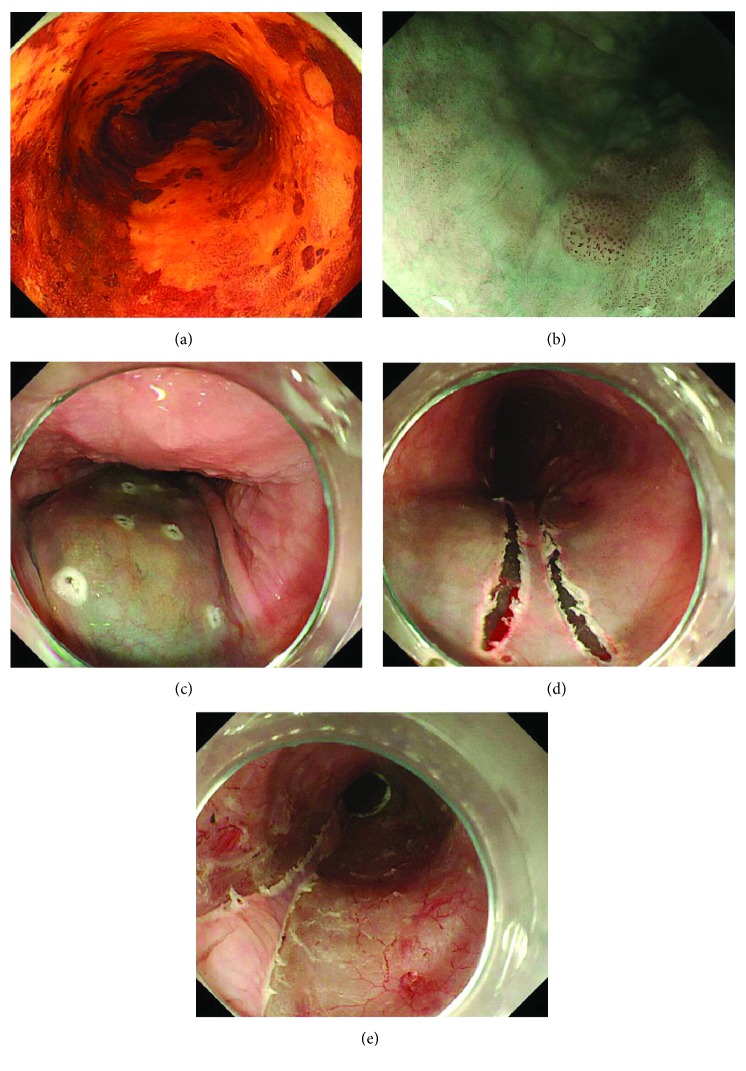
(a) The appearance of subcircumferential superficial esophageal cancer on endoscopy with iodine staining. (b) A B2 or B3 vessel pattern [10, 11] was not recognized around the lesion on magnified narrow band imaging. (c) The boundary of the lesion was marked with a Flush knife. A sufficient volume of glycerol solution was injected into the submucosal layer of the mucosa that was to be preserved. (d) The first incision was made immediately above the markings on both sides of the lesion. (e) There is little damage to the remnant mucosa. The LB method allows for the precise preservation of the mucosa.

Preoperative upper gastrointestinal endoscopy was performed, and the patients in whom a B2 or B3 vessel pattern [10, 11] was recognized around the lesion on magnified narrow band imaging were excluded from the analysis (Figures 1(a) and 1(b)). ESD was performed under general anesthesia. Marking was performed around the boundary of the lesion using a Flush knife. A sufficient amount of glycerol solution was inserted into the submucosal layer of the mucosa that was to be preserved (Figure 1(c)). The first incision was made immediately above the markings on both sides (Figure 1(d)). Trimming was not performed at this time. After making a small incision on the distal side, we connected the incision from the proximal side to the surrounding lesion. A hemoclip with a thread was attached to the proximal side of the specimen, and the thread was pulled out of the mouth. The dissection of the submucosal layer was performed while applying tension to the specimen. The LB method enables the mucosa to be precisely preserved (Figure 1(e)).

### 2.3. Multiple Intraregional Steroid Injection (mISI)

If a mucosal defect covered more than 2/3 of the circumference, mISI was applied. Triamcinolone acetonide (Kenacort, 40 mg/1 mL; Bristol-Myers Squibb, Tokyo, Japan) was diluted 1 : 19 with saline to make a 2 mg/mL solution. A 26-gauge needle was used to inject 2.0 mL (10 injections) of the solution into the residual submucosal tissue of the ulcer bed. If the longer axis of the ulcer bed was >5 cm, then 2.0 mL (20 injections) of the solution was injected. It was easy to inject when the bevel of the needle faced the ulcer bed. The first intraregional steroid injection (ISI) procedure performed just after ESD. ISI was repeated approximately every two weeks until epithelization reached >3/4 of the circumference.

### 2.4. Endoscopic Balloon Dilation (EBD)

Follow-up endoscopy was performed every two weeks. If an endoscope of 9.2 mm in diameter could not pass through the esophagus, EBD was performed using a controlled radial expansion balloon (Boston Scientific, Marlborough, MA, USA). The size of the dilators that was used for the initial procedure ranged from 12 to 15 mm, according to the degree of the stricture.

### 2.5. Statistical Analyses

Fisher's exact test and the Mann–Whitney *U* test were used to evaluate the differences between the groups. The statistical analyses were conducted using the SPSS 15.0 software package (SPSS Inc., Chicago, IL, USA). *P* values of less than 0.05 were considered to indicate statistical significance.

## 3. Results

Eighteen patients and 7 patients were classified into the LB and non-LB groups, respectively. There was no significant difference in the gender or age of the two groups (Table 1). Other comparisons are shown in Table 2. The difference between the median lesion size and the median resected specimen size in the non-LB group was 8.0 mm. On the other hand, the difference in the LB group was only 4.0 mm. The median total triamcinolone dose in the non-LB group was approximately 2.0 times than that in the LB group (non-LB group/LB group: 220.0 mg/106.0; *P* = 0.008).

The ratio of patients with EBD after ESD did not differ between the groups to a statistically significant extent (LB group versus non-LB group: 6/18 patients (33.3%) versus 4/7 (57.1%); *P* = 0.490). However, in the LB group, the median number of endoscopic balloon dilation sessions after ESD tended to be lower than that in the non-LB group (LB group versus non-LB group: 1 versus 2; *P* = 0.122). The median period until complete epithelialization after ESD was significantly shorter in the LB group (LB group versus non-LB group: 51.0 days versus 105 days; *P* = 0.012). The horizontal margins in all 7 patients of the non-LB group were negative. On the other hand, in the LB group, 3 patients (3/18; 16.7%) had unclear margins and 4 patients (4/18; 22.2%) had positive margins. However, there was no local recurrence for approximately two years in either group.

## 4. Discussion

In the present study, the LB method was useful for promoting rapid healing after ESD in patients with subcircumferential esophageal cancer. mISI and the LB method might be useful for preventing esophageal stricture after ESD.

The ESD procedure is widely performed for the treatment of superficial esophageal cancer in Japan; there is no limitation in lesion size if the tumor depth is within the epithelium or the mucosa of the lamina propria [1–4]. However, it is said that the risk of esophageal stricture increases if a mucosal defect covers more than 3/4 of the circumference, especially in full circumference ESD [5–7, 12]. Various approaches, including steroid injection [6, 8], oral steroid administration [9], polyglycolic acid sheet [13, 14], cell sheet [15], collagen patch [16], and stent placement [17, 18], are used for the prevention of the esophageal stricture after ESD. We have previously performed insurance-adapted mISI and are of the opinion that it is safe and effective. However, in our institution, the rate of stricture after ESD in cases in which the wound covered less than half the circumference was 0%; the rate in cases in which the lesion covered less than half the circumference was 7.62%. We assumed that this created too large of a margin around the lesion. Thus, the LB method, which preserves the mucosa to the maximum possible extent, was performed to treat subcircumferential esophageal cancer. The stricture, wound healing, and the results of a pathological examination were compared between the LB group and the non-LB group (in which whole circumference ESD was performed).

With regard to esophageal stricture after whole circumference ESD, the number of patients who required EBD and the number of EBD sessions that were required at our institutions were lower in comparison to other reports [19, 20]. Thus, our mISI method might be effective. At present, there are no standards regarding the number of local injections and the total steroid dose. In our institution, ISI was first performed just after ESD. ISI was repeated approximately every two weeks until the epithelization reached >3/4 of the circumference. The total steroid dose administered to the non-LB group was approximately 2.0 times than that administered to the LB group. The total dose of triamcinolone was greater in comparison to that administered in other facilities [6, 8, 20], which probably had a good effect on the prevention of stricture. We hypothesize that our mISI method had a preventive effect against stricture after whole circumferential ESD. However, in order to investigate this hypothesis, it would be necessary to compare our mISI group to a group of patients undergoing whole circumferential ESD in whom the steroid dose was restricted. Moreover, the ratio of patients with EBD after ESD did not differ between our two groups to a statistically significant extent. If the number of patients increases, it might prove the preventive effect of the LB method against stricture.

On the other hand, the period until complete epithelization in the LB group was significantly shorter than that in the non-LB group. Epithelialization after ESD occurs from the margin of the normal mucosa. Thus, it is easy to imagine that the LB group, in which much of the mucosa is preserved, will heal earlier than the non-LB group. However, the administration of steroids might prolong epithelialization. The possibility that healing was significantly delayed in the non-LB group cannot be denied. However, it is thought that the amount of steroid used in non-LB method was necessary to prevent esophageal stricture after ESD. Moreover, the LB method was considered to contribute to shortening the period until complete epithelization under the condition of steroid administration, which minimized the occurrence of stricture after ESD.

The pathological evaluation of resected specimens revealed that the rate of curative resection was 100% (7/7) in the non-LB group and 61.1% (11/18) in the LB group. No residual lesions were on follow-up endoscopy for approximately two years. Based on these results, the procedures in all of the cases in the LB group might finally be considered to be clinically curative. However, follow-up endoscopy should be carefully performed every 3–6 months in cases in which resection is noncurative and additional treatments, such as mucosal resection or argon plasma coagulation should always be prepared.

We experienced one case in which a patient underwent whole circumferential ESD for a whole circumferential lesion and in whom additional chemoradiotherapy was considered to be necessary. The esophageal ulceration had still not completely healed at 4 months after ESD. He could not wait until such ulceration had completely healed. Therefore, he received chemoradiotherapy which thus led to the onset of severe stricture due to radiation. Thus, whole circumferential ESD plus local steroid injection may delay the start of additional chemoradiotherapy. In other words, whole circumferential ESD can be tolerated when the possibility of additional treatment is low or when surgery for additional treatment is desired. However, if additional chemoradiotherapy is desired, the esophageal ESD should be limited to lesions down to the subcircumference (within the indication of the LB method).

This study is associated with several limitations. It was a retrospective study that was performed in a single institution. Therefore, the number of patients was small. In addition, there was a significant difference in the steroid doses that were administered to the two groups.

In conclusion, the LB method plus mISI is useful for promoting rapid healing after ESD in cases of subcircumferential superficial esophageal cancer and might have a preventive effect against esophageal stricture.

## Figures and Tables

**Table 1:** The patients' characteristics.

	Non-LB group	LB group	*P* value
Circumference of mucosal defect	1	5/6 < < 1	
Number of patients	7	18	
Male/female	5/2	14/4	0.775^※^
Age median (years) [range]	72.0 [55–80]	74.0 [49–85]	0.442^※※^
Tumor location	Ut 2, Mt 4, Lt 1	Ut 4, Mt 11, Lt 3	0.782^※※^

Ut: upper thoracic esophagus; Mt: middle thoracic esophagus; Lt: lower thoracic esophagus; ^※^Fisher's exact test; ^※※^Mann–Whitney *U* test.

**Table 2:** The patients' characteristics and the details of management after ESD.

	Non-LB group	LB group	*P* value
Number of patients	7	18	
Median procedure time (min) [range]	156.0 [110–210]	140.0 [60–340]	0.544^※※^
Median lesion size (mm) [range]	59.5 [52–85]	56.0 [42–82]	0.412^※※^
Median resected specimen size (mm) [range]	67.5 [59–105]	60.0 [47–87]	0.363^※※^
pHM x (%)	0 (0)	3 (16.7)	0.724^※^
pHM1 (%)	0 (0)	4 (22.2)	0.558^※^
Local recurrence (%)	0 (0)	0 (0)	
Median observation period (days) [range]	806 [184–2512]	763 [42–1834]	0.288^※※^
Median post-ESD period until complete epithelization (days) [range]	105 [48–132]	51.0 [25–76]	0.012^※※^
Number of EBD patients (%)	4 (57.1)	6 (33.3)	0.490^※^
Median number of EBD sessions (number) [range]	2 [2–9]	1 [1–8]	0.122^※※^
Median total Triamcinolone dose (mg) [range]	220 [152–312]	106 [40–216]	0.008^※※^
Median injection sessions (number) [range]	5.0 [2–10]	2.3 [1–4]	0.025^※※^

^※^Fisher's exact test; ^※※^Mann–Whitney *U* test.

## Abbreviations

ESD: Endoscopic submucosal dissection
mISI: Multiple intraregional steroid injection
LB: Log bridge
EBD: Endoscopic balloon dilation.
